# Supplementary material for: Effect of Overexpression of γ-Tocopherol Methyltransferase on α-Tocopherol and Fatty Acid Accumulation and Tolerance to Salt Stress during Seed Germination in Brassica napus L
Source: Int J Mol Sci. 2022 Dec 14;23(24):15933. doi: 10.3390/ijms232415933 (PMC9784450; doi:10.3390/ijms232415933)
Supplement: Supplementary file 1 [file ijms-23-15933-s001.zip › ijms-1968401 - supplementary.pdf]

International Journal of *Molecular Sciences*

Supplementary Materials:

Table S1. The primers used in this study.

Primer Name	Orientation	Sequence (5′-3′)	Annotations
BnaC02.TMT.a_Fc	Forward	AATCCCATACACAGCCACA	specific primers for
BnaC02.TMT.a_Rc	Reverse	CTAAAATAGACAACGTAGCC	*35S:BnaC02.TMT.a-6HA*
BnaC02.TMT.a-Xhol-F	Forward	CCGCTCGAGATGAAAGCGACTCTCGCAC	nested primers for
BnaC02.TMT.a-Cla1-R	Reverse	CCATCGATGAGAGGCTTCTGGCAAGTG	*35S:BnaC02.TMT.a-6HA*
35S_P	Forward	GACCCTTCCTCTATATAAGGAAGTTC	DNA genotyping
BnTMT_R	Reverse	GAGAGGCTTCTGGCAAGTG	
BnGAPDH_F-qPCR	Forward	CCGCTTCCTTCAACATCATT	RT-qPCR
BnGAPDH_R-qPCR	Reverse	GTCGCAGCTTTCTCGAGTCT	
BnTMT_F-qPCR	Forward	GCCTCCCAAGGCACAAAGT	
BnTMT_R-qPCR	Reverse	TCCGCTATTCCTTCTCGCAG	
BnFAD2_F-qPCR	Forward	GTTTCCTCGTGTTGATCACTTAC	
BnFAD2_R-qPCR	Reverse	TGATAATGCGGCATCGTGGA	
BnFAD3_F-qPCR	Forward	TATAAGGGCGGCCATTCCTAAGCA	
BnFAD3_R-qPCR	Reverse	AGATAGCCCAGAACAGGGTTCCTT	

Table S2. The standard curve of α-, β-, γ-, and δ-tocopherol.

Tocopherol Forms	Standard Curve	Linearity Range (μg/mL)	Retention Time (min)
α-tocopherol	$y = 4.3411x - 3.7525$; $R^2 = 0.9996$	0.4~200	12.445
β-tocopherol	$y = 6.2545x - 0.6437$; $R^2 = 0.9999$	0.4~200	11.209
γ-tocopherol	$y = 6.7402x - 3.6822$; $R^2 = 0.9996$	0.4~200	10.771
δ-tocopherol	$y = 4.9310x - 1.0052$; $R^2 = 0.9998$	0.4~200	9.443

Percent Identity

Divergence	1	2	3	4	5		
1		89.3	89.2	88.4	89.5	1	Atγ-TMT (NP_176677.1)
2	11.6		98.3	98.6	98.0	2	BnaC02.TMT.a (BnaC02T0331100ZS)
3	11.6	1.8		97.4	98.8	3	BnaC02.TMT.b (BnaC02T0197500ZS)
4	12.6	1.5	2.6		96.5	4	BnaA02.TMT.a (BnaA02T0247300ZS)
5	11.3	2.1	1.2	3.6		5	BnaA02.TMT.b (BnaA02T0154300ZS)
	1	2	3	4	5		

Figure S1. Percent identity (up) and divergence (down) of the amino acid sequences of the γ-TMT protein from *A. thaliana* and *B. napus*. 1: Atγ-TMT (NP_176677.1); 2: BnaC02.TMT.a (BnaC02T0331100ZS); 3: BnaC02.TMT.b (BnaC02T0197500ZS); 4: BnaA02.TMT.a (BnaA02T0247300ZS); 5: BnaA02.TMT.b (BnaA02T0154300ZS). Divergence and percent identity were calculated by comparing sequence pairs to the phylogeny reconstructed by MegAlign.

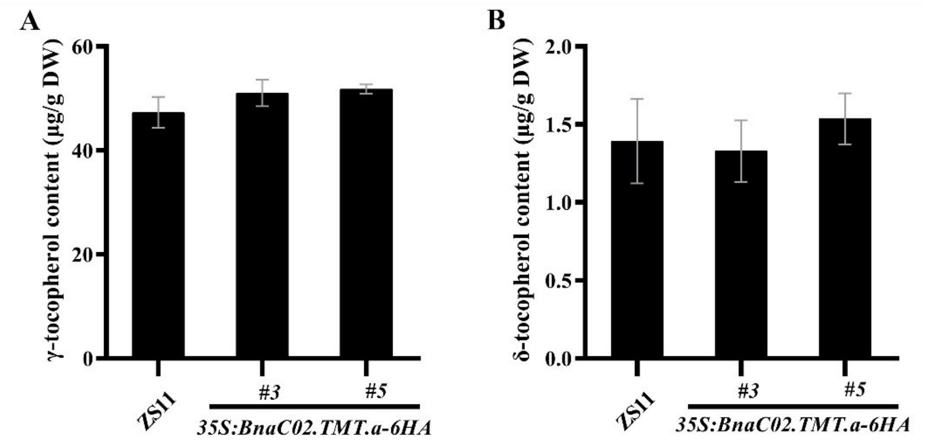

Figure S2. γ-tocopherol (A) and δ-tocopherol (B) content of wild-type ZS11 and *35S:BnaC02.TMT.a-6HA* plants in mature seed of rapeseed. Error bars denote standard deviation.

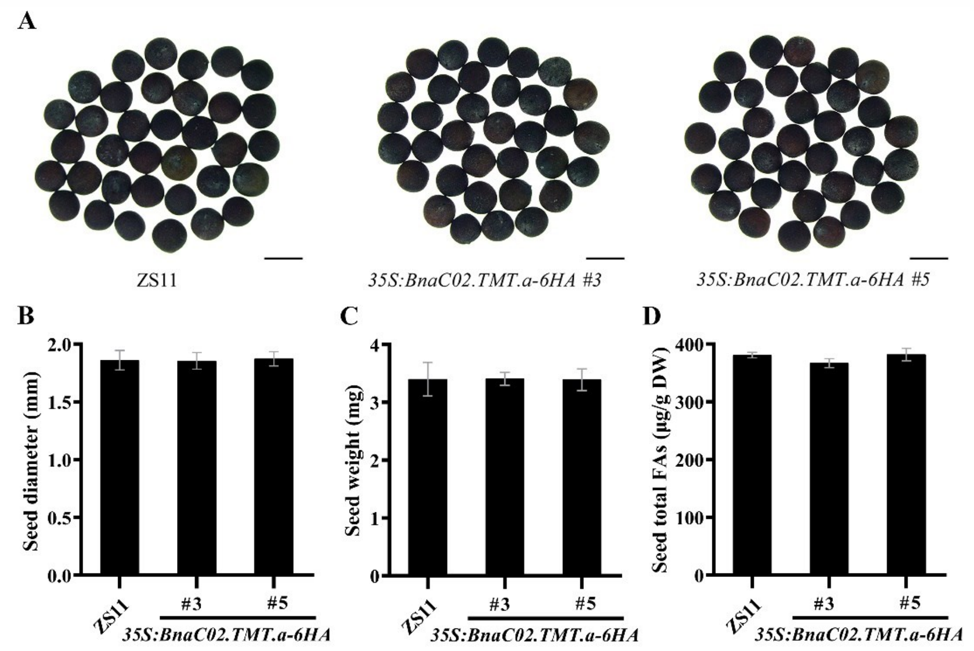

Figure S3. Analysis of seed coat color, seed size, seed weight and seed total FAs among wild-type ZS11 and *35S:BnaC02.TMT.a-6HA* plants. (A) Microscopic observations of randomly selected mature seeds. Bars = 2000 μm. (B) Quantitative comparisons of seed size (length and width) between ZS11 and *35S:BnaC02.TMT.a-6HA* plants. (C) Quantitative comparisons of seed weight between ZS11 and *35S:BnaC02.TMT.a-6HA* plants. In (B) and (C), Values represent means ± SD (n = 5), and each of the three assays for each biological replicate contained 200 seeds from 8 individual plants grown in different pots arranged randomly within one of three blocks. (D) Seed total FA content in seeds among ZS11 and *35S:BnaC02.TMT.a-6HA* plants.

```
BnaC02.TMT.a(BnaC02T0331100ZS)  MKATLAPPSSLISLPRHKVSSLRSPSLLLQSQRPSSALM-TTTASRGSVAVTAAATSSAE  (59)
BnaA.VTE4.a1(EU637012.1)        MKATLA-PSSLISLPRHKVSSLRSPSLLLQSQRPSSALMTTTTASRGSVAVTAAATSSFE  (59)
                                      *                                *                  *

BnaC02.TMT.a(BnaC02T0331100ZS)  ALREGIAEFYNETSGLWEEIWGDHMHHGFYDPDSSVQLSDSGHREAQIRMIEESLRFAGV  (119)
BnaA.VTE4.a1(EU637012.1)        ALREGIAEFYNETSGLWEEIWGDHMHHGFYDPDSSVQLSDSGHREAQIRMIEESLRFAGV  (119)

BnaC02.TMT.a(BnaC02T0331100ZS)  TEEEKKIKRVVDVGCGIGGSSRYIASKFGAECIGITLSPVQAKRANDLAAAQSLSHKVSF  (179)
BnaA.VTE4.a1(EU637012.1)        TEEEKKIKRVVDVGCGIGGSSRYIASKFGAECIGITLSPVQAKRANDLAAAQSLSHKVSF  (179)

BnaC02.TMT.a(BnaC02T0331100ZS)  QVADALDQPFEDGIFDLVWSMESGEHMPDKAKFVKELVRVTAPGGRIIIVTWCHRNLSQG  (239)
BnaA.VTE4.a1(EU637012.1)        QVADALEQPFEDGIFDLVWSMESGEHMPDKAKFVKELVRVAAPGGRIIIVTWCHRNLSPG  (239)
                                      *                                 *                 *

BnaC02.TMT.a(BnaC02T0331100ZS)  EESLQPWEQNLLDRICKTFYLPAWCSTSDYVELLQSLSLQDIKCADWSENVAPFWPAVIR  (299)
BnaA.VTE4.a1(EU637012.1)        EEALQPWEQNLLDRICKTFYLPAWCSTSDYVDLLQSLSLQDIKCADWSENVAPFWPAVIR  (299)
                                  *                            *

BnaC02.TMT.a(BnaC02T0331100ZS)  TALTWKGLVSLLRSGMKSIKGALTMPLMIEGYKKGVIKFGIITCQKPL  (347)
BnaA.VTE4.a1(EU637012.1)        TALTWKGLVSLLRSGMKSIKGALTMPLMIEGYKKGVIKFGIITCQKPL  (347)
```

Figure S4. Alignment of protein sequences of BnaC02.TMT.a from *B. napus* 'ZS11' and BnaA.VTE4.a1 from *B. napus* 'Express'. The alignment was conducted using the MUSCLE program (http://www.ebi.ac.uk/Tools/msa/muscle/) and the different amino acids are indicated by asterisks. S-adenosylmethionine binding sites are underlined.
